# Effects of propolis-loaded nanoliposomes fortification in extender on buffalo semen cryopreservation

**DOI:** 10.1038/s41598-023-37424-2

**Published:** 2023-06-30

**Authors:** Sameh A. Abdelnour, Mahmoud A. E. Hassan, Ahmed. M. Shehabeldin, Mohamed. E. A. Omar, Wael A. Khalil, Reham Mokhtar Aman

**Affiliations:** 1 Department of Animal Production, Faculty of Agriculture, Zagazig University, Zagazig, 44511 Egypt; 2 Agriculture Research Centre, Animal Production Research Institute, Ministry of Agriculture, Dokki, Giza, 12619 Egypt; 3 Department of Animal Production, Faculty of Agriculture, Mansoura University, Mansoura, 35516 Egypt; 4 Department of Pharmaceutics, Faculty of Pharmacy, Mansoura University, Mansoura, 35516 Egypt

**Keywords:** Developmental biology, Zoology

## Abstract

Buffalo sperm is sensitive to cryoinjuries, thus improving sperm cryoresistance is a critical approach for wide spreading the assisted reproductive technologies in buffalo. The intention of this work was to assess the effect of propolis-loaded in nanoliposomes (PRNL) supplementation of semen extender on semen quality, antioxidant status and some apoptotic genes of cryopreserved buffalo semen. PRNL were prepared using cholesterol (Chol) as well as soybean lecithin and their physicochemical properties were characterized. Egyptian buffalo bulls (4–6 years) were involved, and the semen samples were collected using the artificial vagina method. Buffalo semen was pooled (n = 25 ejaculates) and cryopreserved in tris extender containing PRNL at 0 (PRNL0), 2 (PRNL2), 4 (PRNL4) and 6 µg/mL (PRNL6), respectively. The PRNL had a size of 113.13 nm and a negative zeta potential (− 56.83 mV). Sperm progressive motility, viability, membrane integrity, abnormalities, chromatin damage, redox status, apoptosis status, and apoptotic genes were investigated after post-thawed buffalo semen. Using 2 or 4 µg/mL PRNL significantly increased sperm progressive motility, viability, and membrane integrity, while sperm abnormalities and the percentage of chromatin damages were the lowest in PRNL2 group. Moreover, the PRNL2 group exhibited the best results for all antioxidative activities (TAC, SOD, GPx and CAT) with significantly higher levels than the other groups (*P* < 0.05). The levels of ROS and MDA were significantly lower in the PRLN2 compared with other groups. The sperm caspase 3 enzyme activities showed the lowest values in PRNL2 groups followed by PRNL4 and PRNL6 groups with significant differences compared with the control. Adding 2 µg/mL PRNL to freezing media significantly reduced apoptotic genes such as *Bax* and *Caspase 3* in sperm, while significantly increase in *Bcl2* expression compared with the control (*P* < 0.001). The expression of *Bcl2, Caspase 3* and *Bax* genes in sperm were not affected by the 6 µg/mL PRNL addition (*P* > 0.05). The electron micrography descriptions exemplified that the fortification of 2 or 4 µg/mL PRNL maintained the acrosomal and plasma membrane integrities as well as sustained the ultrastructure integrity of the cryopreserved buffalo spermatozoa when compared with control group, whereas the 6 µg/mL of PRNL demonstrated highest injury to the acrosome and plasma membranes. Results show supplementation of the buffalo freezing extender with 2 or 4 µg/mL of PRNL enhanced post-thawed sperm quality via boosting the antioxidant indices, diminishing the oxidative stress and apoptosis as well as maintained the ultrastructure integrity of frozen-thawed buffalo sperm.

## Introduction

Cryopreservation of sperm has become a prevalent method for the ongoing semen preservation of genetically merit animals, associated with transgenic lines, and mammalian threatened species^1^. Likewise, sperm cryopreservation supports the wide spread of genetic diversity, and contributed significantly into the development of reproductive technologies globally, such as embryo transfer, IVF, and artificial insemination. The sperm is sensitive to temperature changes, and the successful sperm cryopreservation depending on several factors such as extension, chilling, freezing and thawing protocols^2,3^. The most critical hurdle during the sperm cryopreservation is the generation of excess amount of oxidative stress (OS), which resulted in reducing semen quality^1^. Due to the high amount of PUFAs (polyunsaturated fatty acids) in buffalo’s plasma membrane, make it very extremely vulnerable to OS. However, the over-formed reactive oxygen species (ROS) persuaded by those previous procedures during cryopreservation can interruption the equilibrium between antioxidant defense and ROS production, accordingly, leading to multiple disturbances of sperm functionality and possibly reduced fertility^2^. Previous works demonstrated that several phytochemicals^4^ or its nanoform^1,5^ have been widely used in freezing semen extenders to maintain sperm structure and functionality and diminish the harmful effects of ROS triggered by cryopreservation^3,6^.

Nanoencapsulation is a promising scheme to boost the solubility, bioavailability and stability of bioactive molecules as well as their conceivable purposes in different scientific fields^3^. Liposomes are spherical vesicles consisting of biodegradable and biocompatible phospholipids representing biological membranes^1,3^. Nanoliposomes (NLPs) are one of the utmost attractive nanocarriers, with very small particle, owing to their benefits such as biocompatibility, flexibility, amphiphilicity, efficient cellular uptake, non-toxicity, and non-immunogenicity^7,8^. Moreover, NLPs have been applied for the encapsulation of natural pigments, enzymes, fatty acids, vitamins, plant extracts and their essential oils^7,9^. Recently, natural nano molecules are reported to have several biological activities and are widely fortified in freezing media to enhance semen cryopreservation^2^ due to their safety, and therapeutic possessions.

Propolis is a natural resinous constituent synthesized by bees that has been extensively applied as a folk medicine since ancient times. Numerous compounds have been recognized in propolis, such as caffeic acid, gallic acid, quercetin, chrysin, galangin and pinocembrin^10,11^. Moreover, propolis has been presented to demonstrate strong biological actions, such as antibacterial, antiviral, anticancer, and antioxidant properties^12^. Addition of propolis to semen ram extender (0.5 and 1.0 mg/mL; preserved at 5 °C for 48 h) enhanced sperm function, and antioxidant capacity as well as reduced the lipid peroxidation^13^. Moreover, several other studies shown a significant enhance in semen longevity in rabbits^14^, and ram^15^ by propolis administration. Additionally, propolis was add to the cryopreserved semen media in fish^16^, goats^17^, bull^11^, ram^12^. The cryoprotective effect of propolis could be due to its ability to promote antioxidant events and significantly decreased the percentages of apoptosis sperm^11,12^. Certain trials anticipated that the constructive impact of propolis on sperm quality and functionality could be accredited by its favorable unrivaled structural molecules permitting the protection of sperm cells. Al-Nawab et al.^10^ found that the using of propolis extract (0.5–1 mg/ mL) has a considerable consequence on in vitro enhancement of sperm motility and health in infertile patient via maintaining the sperm membrane and DNA integrity as well as support the mitochondria function. Many studies reported that different natural compounds coated with liposome showed significant influence on cryopreserved semen function in animals^3,7–9^. However, the use of propolis-loaded nanoliposomes (PRNL) fortified with freezing media of buffalo semen has previously been unexplored. Consequently, the target of the existing research was to assess the efficacy of cryoprotective effect of PRNL as antioxidant and anti-apoptosis mediators in cryopreserved buffalo semen.

## Material and Methods

### Materials

Chinese propolis (PR) was acquired from ABChem Company (Mansoura, Egypt). Chol was obtained from Loba Chemie Pvt. Ltd. (Mumbai, India). The SL (soybean lecithin) was procured from Carlo Erba Reagents (Chaussée du Vexin, France). From Cornell lab (Cairo, Egypt), both methanol and chloroform (HPLC grade, Fischer) were procured.

### Preparation of PRNL and PR-free nanoliposomes (NL_free_)

The traditional thin-film hydration procedure was adopted in the preparation of PRNL, as delineated previous, with minor amendments^18^. In brief, accurately weighed quantities of PR, Chol and SL, 10, 20 and 380 mg, respectively, were dissolved in 10 mL of the organic solvent mixture (methanol and chloroform, 1:2 *v/v*). In a 250 mL rounded-bottom flask and using a rotary evaporator (Heidolph LABOROTA 4000, Serial No. 030002422, Germany), the organic solvent mixture was fumigated under vacuum into a thin lipid film. Where, the temperature was adjusted to 60 °C (Heidolph Heizbad WB, Serial No. 020004125, Germany), whilst the speed of rotation was fixed to 70 rpm untill full the evaporation of the organic solvent mixture. To have a crude nanoliposomal suspension, the obtained transparent lipid membrane was hydrated with DW (deionized water; 20 mL) at 60 °C and 120 rpm, for 20 min. Thereafter, to yield one-phase NLPs, the suspension was homogenized using an ultrasonic probe (Serial No. 2013020605, Model CV 334) involved to a homogenizer (Sonics Vibra-cell™, Model VC 505, Sonic & Materials, INC., USA) and following the succeeded circumstances: (Pulser: 1 s ON/1 s OFF, Timer: 3 min, Amplitude: 90%,), in an ice bath. Eventually, the attained NLPs squanderings were deposited at 4 °C until usage. For preparation of the conforming NL_free_, without PR in the organic solvent mixture, the same procedures were adopted.

#### Physicochemical characterization of PRNL

##### Determination of PR entrapment efficiency percent (EE %)

Indirectly, entrapment efficacy percentage (EE %) of PR was assessed. Unentrapped PR was detached from the synthesized PRNL via centrifugation process at 10.000 rpm for 60 min (ACCULAB Cooling centrifuge, CE16-4X100RD, USA) using Amicon®, 4 mL and 10 KDa cutoff units, Ultra-4 Centrifugal Filter Units (Merck Co., California, USA).

Afterwards, free PR amount in the ultrafiltrate was determined spectrophotometrically against the ultrafiltrate of NL_free_ as a blank, at 296 nm (ultraviolet/visible (UV–VIS) double beam spectrophotometer, Labomed Inc., USA). Next, pre-studied spectrum of PR revealed that the optimum absorption peak was at 296 nm. Therefore, the wavelength (296 nm) was utilized to concept a calibration curve that was indicated according to obey Beer–Lambert’s Law. The EE % was intended rendering to the following Eq. (1):

1 $$EE \%=\frac{{\mathrm{PR}}_{total }-{\mathrm{PR}}_{free}}{{\mathrm{PR}}_{total }}\times 100$$

Moreover, after centrifugal concentration, both PRNL and NL_free_ were washed three times with DW, then resuspended in DW, and ultimately were subjected to freeze-drying (SIM FD8-8 T, SIM international, USA). Lastly, the freeze-dried specimens were maintained at 4 °C for further supplementary description.

##### ζ-potential (ζP), particle size distribution (PDI) and hydrodynamic diameter (D_h_) measurements

The ζP, D_h_, and PDI values of the freshly synthesized PRNL were assessed by a Zetasizer Nano ZS (Malvern Instruments, Malvern, UK). The rate ζP was evaluated by the Laser Doppler Electrophoresis system, which measures the electrophoretic mobility of the NPs in an electrical field, while D_h_ and PDI were evaluated utilizing the DLS (dynamic light scattering) procedure. In triplicates, all the aforesaid measurements were carried out, after advisable dilution of NLPs with DW, at room temperature.

##### Transmission Electron Microscopy (TEM)

The TEM is a system that allows capture of NPs' images with very high tenacity. A morphological investigation of the freshly prepared PRNL was probed by TEM (JEOL JEM-2100, Tokyo, Japan). An aliquot of NLPs dispersion was extended with DW, and then 20 µL of the mixed specimens were loaded onto a carbon-coated copper grid, permitted 10 min at room temperature for drying and immediately scrutinized via TEM without any staining. Finally, the image analysis and acquisition procedures were supported via Soft Imaging Viewer software (Gatan Microscopy Suite Software, version 2.11.1404.0) and Digital Micrograph.

##### Fourier transform-infrared spectroscopy (FT-IR)

The FT-IR can be considered as a supplementary technique that depicts functionalization and vibrational description of the synthesized NLPs, where it manifests the signatory vibrational peaks of each component. Moreover, the infrared spectra of PR, Chol, SL, the corresponding physical mixture to the synthesized NLPs formula (exactly weighed amounts of 10 mg PR, 20 mg Chol and 380 mg SL), alongside freeze-dried medicated NLPs, namely; PRNL, and its comparable NL_free_ were developed by a FTIR Spectrophotometer (Bruker Alpha II Platinum, Billerica, MA 01,821, USA) over a range of 500 to 4000 cm^−1^, applying the attenuated total internal reflectance (ATR) technique.

##### Comparative stability studies of PRNL

To assure the efficacy and safety of drug products, stability evaluation has become crucial in the pharmaceutical field. To accomplish such necessary appraisal, the freshly prepared PRNL suspensions were filled into glass bottles with air-tight closures and the stability screening was fulfilled at long-term storage environments (4 ± 1 °C) as well as accelerated storage environments (25 ± 1 °C, 60 ± 5% RH) for 3 months^19^. The NLPs were estimated, initially (freshly synthesized NLPs at construction day as earlier depicted) and at itemized time intervals (1 and 3 months), as regards prudent forefingers for their kinetic stability specifically; physical appearance, ζP, D_h_, and PDI. Moreover, drug retention (%) was calculated as follows:

2 $$ Drug\;retention \left( \% \right) = \frac{EE \;\% \;at\; each \;time\;interval}{{ initial\;EE \% }} \times 100 $$

### Ethical statement

The investigational fulfillments in this research that complicated animals were permitted by the IACUC (Institutional Animal Use and Care Committee), Zagazig University (Approval number, ZU IACUC/2/F/173/2022). According to the ARRIVE guidelines, the use and care of animals were totally followed those rules.

### Animals and semen collection

Five healthy Egyptian buffalo bulls (4–6 years) with higher sexual behavior and proven with good fertility (based on records and semen assessments) were utilized for this experiment. The animals were reared at the ILMTC (International Livestock Management Training Center), Sakha, Province of Kafr El-Sheikh, Agriculture Research Center, Egypt. The bulls were reared at the ILMTC under the same conditions of management, feeding, and environmental that used for semen collection. The animals were fed with concentrate diet containing crude protein (21%) and total digestibility nutrients (70%) and clover hay as a forage diet. The drinking water was provided free access to fresh and clean. All methods were performed in accordance with the relevant guidelines and regulations by IACUC (Institutional Animal Use and Care Committee), Zagazig University.

Semen samples (October and November months, 2022) were routinely picked up weekly for successive 5 weeks via artificial vagina method at 42 °C. Next, the specimens were transported to laboratory and preserved at 37 °C in water bath for initial evaluations. The ejaculates (twenty five ejaculates) of individual bulls were assessed for volume, motility, abnormalities sperm account. Additionally, the succeeding lowest values with at least (70%) motility, volume (5 mL), abnormalities (15%) and sperm concentration 500 × 10^6^ sperm/mL were included^20^. After each semen collection, semen samples were mixed for making pooled semen and used for the present experiment.

### Freezing–thawing procedures

According to the ILMTC protocols, the freezing extender used in the current experiment was contained Tris (3.028 g/dL), citric acid (1.675 g/dL), fructose (1.25 g/dL), 20 mL of egg yolk, 6.0% of glycerol and antibiotics [streptomycin (100 µg/mL) and penicillin (100 IU/mL)]^21^. The prepared freezing media was diluted with the dilution rate 1 semen: 10 extenders. The pooled extended semen samples were supplemented with three levels of propolis-loaded nanoliposomes (PRNL); 2 µg/mL (PRNL2), 4 µg/mL (PRNL4), and 6 µg/mL (PRNL6), as well as the control group (PRNL0; without any supplementation). The semen extended samples were automatically loaded into 0.5 mL French mini straw (with final sperm concentration 50 × 10^6^ sperm/mL), and equilibrated at 5 °C for 4 h. Then, the semen straws were placed 5 cm overhead the liquid nitrogen for 10 min. followed by dropping into liquid nitrogen (− 196 °C) and deposited pending thawing process.

Semen thawing was realized at 37 °C for 30 s in a water bath^21^. The extender composition and freezing and thawing protocols were focused conferring to ILMTC guidelines and our earlier work^21^. After thawing, the post-thawed sperm were evaluated for progressive motility, viability, membrane integrity, abnormality, and chromatin damage. The redox status in sperm were also evaluated including, Catalase (CAT); Glutathione Peroxidase (GPx); Reactive Oxygen Species (ROS); Total Antioxidant Capacity (TAC); Supeoxide Dismutase (SOD) and Malondialdehyde (MDA).

### Post-thaw sperm assessment

#### Sperm motility

Sperm motility was determined by insertion a droplet of the post-thawed semen sample on a pre-tempered slide (37 °C) with a coverslip and detecting the sample with a phase-contrast microscope (400× magnification). Then, the sperm were detected in at minimum various fields (5 views) on a thermostatically controlled point sustained at 37 °C and stated as proportion progressive motility with validity value around ± 5%.

#### Sperm viability

Viability is a reflection of the percentage of live sperm detected by the assessment of cellular veracity. According to the method of^22^, the sperm viability (%) was measured using the staining of Eosin-Nigrosin technique. Briefly, 10 µL of post-thawed semen positioned on a pre-warmed glass slide and mixed slightly with 20 µL of Eosin-Nigrosin stain, then left in air drying condition at 32–34 °C for 2 min. At least, sperm (200/sample) were calculated in duplicates in a slight ready with a light microscope (400× magnification), for dead (pink or stained) or live (unstained/non-eosinophilic) sperm.

#### Plasma membrane integrity

The hyposmotic swelling (HOS) technique was used for detecting the sperm plasma membrane integrity. Concisely, post-thawed semen (50 µL) was mingled with 500 µL of HOS solution [fructose (1.35 g) and sodium citrate (0.73 g) were dissolved in 100 mL DW; osmolality 150 mOsm/kg) and incubated at 37 °C for 30 min. Next, the semen samples were transported to a glass slide, shielded with a cover slip, and scrutinized under a phase-contrast microscope (400×; Olympus BX20, Tokyo, Japan). At least, spermatozoa (two hundred/sample) were evaluated for swelling categories. Sperm with interrupted membranes show straight tails while those with intact plasma membranes show coiled or swollen tails^20^.

#### Evaluation of sperm abnormalities

Using the Hancock's solution, and followed the method of Schäfer et al.^23^, sperm morphology were assessed. A 5 μL of thawed semen samples were mixed with 1 mL Hancock’s solution in sterilized centrifuge tube. After that, 10 µL of mixed semen samples were positioned on a pre-warmed slide, and the proportion of all sperm abnormalities (including heads, tails, and mid-pieces) were documented by calculating an entire of two hundred sperm using a phase-contrast microscope (× 1000).

#### Chromatin damage

After thawing process, thawed semen was washed several times with phosphate buffered saline (PBS). Then, the semen was centrifuged, and sperm pellet was re-suspended in 0.5 mL of PBS. Semen samples (50 µL) were suspended and then glass smeared. A 3 smears from each sample were arranged on sterilized slides, drying and then fixed with Carnoy’s solution (methanol/acetic acid, 3:1) and kept for overnight according to Liu and Baker^24^. Once samples cleaned and dried, the slides were subjected to staining for five min with freshly prepared acridine orange staining (AO) as follows: AO (10 mL, 1%) in distilled water was fortified to a blend of citric acid (40 mL 0.1 M) and Na_2_HPO_4_7H_2_O (2.5 mL, 0.3 M). Previously, the AO solution was prepared and deposited in a dark condition at 4 °C for one month. After washing several times, then subjected to drying, the slides were scrutinized via a fluorescent microscope (excitation of 450–490 nm; Leitz, Germany). Sperm with DNA integrity, or intact chromatin had green fluorescence, while sperm with an irregular DNA produced fluorescence in a spectrum fluctuating from red to yellow green.

### Assessment of antioxidant activities

Assessing the oxidative stress and antioxidant indices are critical for semen used in artificial insemination. The samples of post-thawed semen were subjected to centrifugation (1500 g for 15 min at 4 °C, Sigma 2–16 KL), thenceforth seminal plasma was detached and deposited at − 20 °C. The antioxidant profile in cryopreserved semen supplemented with different does of PRNL was scrutinized by spectrophotometrically method. The TAC, SOD, CAT, and GPx activities^25^ and oxidative stress including MDA and ROS levels were assessed by commercially ELISA kits (Beyotime Biotechnology, Shanghai, China) following the manufacturer’s instructions. The methods for detecting the previous parameters have been descried in detail of the technique^20,26^.

### Sperm Caspase 3 activity

Caspase-3 enzyme activity was defined in all post-thawed sperm in treated and untreated groups based on the method of Taylor et al.^27^. A spectrophotometric detection assay based on of the chromophore p-NA (p-nitroaniline) after cleavage from the labeled substrate DEVD-p-NA. A 100 µL of cell suspension (1–5 × 10^6^) cell/mL was prepared and suspended in PBS. Then, semen samples were resuspended in chilled Cell Lysis Buffer (50 µL) and then the cells incubated on ice for 10 min. Semen samples were washed with PBS/BSA (Bovine serum albumin, 1:1 dilution rate) with 2 mL and then centrifuge at 2000 g for 5 min. after compete centrifuge, the supernatant was ignored, and pellet cells were resuspended in PBS washing solution (100 µL). A 10 µL of caspase 3 (Rabbit anti-active caspase 3 cat; No. 559341, Abcam, UK) was blended well with pellet sperm then the mix was incubated at 37 °C for 30 min in dark condition. Finally, cells were resuspended in 200 µL of 4%paraformaldehyde in PBS and then sperm fixed until acquired by flow cytometry.

### Sperm ultrastructure

According to prior studies^21,28^, sperm ultrastructure was evaluated using TEM. Briefly, post-thawed semen samples (500 μL) were centrifugated and resuspended in a fixative solution (4% glutaraldehyde in Dulbecco’s modified PBS at 4 °C for 2 h). Then, semen samples were washed several times and post-fixed in 1% of osmium tetroxide at RT for 1 h. The fixed specimens were exposed to dehydration in gradual ethanol, then preserved with propylene oxide, and embedded in ultrathin and Epon resin, then sectioned (60–70 nm) for TEM. Ultrathin segments were detected at 80 kV via a TEM (JEOL 2100 TEM at 80 kV). Where, the changes in sperm ultrastructure in each treatment were observed at the head region changes for scrutinize the states of acrosome, chromatin, nucleus, and plasma membrane. The mid-piece was explored for observing the changes in sperm mitochondria morphology.

### Total RNA extraction and *mRNA* quantification

Total RNA was separated from post-thawed cryopreserved spermatozoa using TRIzol reagent (Invitrogen Co., USA) subsequent the producer’s directions, and the quality and concentration were ascertained using a Nanodrop spectrophotometry (Nanodrop 2000, Thermo Fischer, CA, USA), with absorbance ratio was A260/280. The cDNA was produced via a PrimeScript RT Reagent Kit (Takara, Dalian, China) as indicated in the attached pamphlet. The RT-qPCR was employed to inspect the expression of pro-apoptotic Bax (BCL2-associated X), anti-apoptotic Bcl-2 (B-cell lymphoma-l), apoptotic caspase 3 and the β-actin genes was used as a housekeeping gene (Table 1). RT-qPCR assay was accomplished via the ChamQ ™ SYBR qPCR Master Mix (Vazyme Biotech, Nanjing, China), and reactions were achieved as follows: 95 °C for 30 s, 40 cycles of 95 C for 5 s followed by 60 °C for 30 s. Using the 2^−ΔΔCt^ technique^29^, the comparative gene transcript levels were investigated with β-actin as a housekeeping gene.

**Table 1:** Primers of genes used in the current trial (*Bax, Bcl-2, Caspase-3*, and *β-actin)* for real time PCR.

Gene	Sequence (5′–3′)	Amplicon size (bp)
*Caspase-3*	**F:** 5′-GGACTGTGGCATTG AGACAG-3′	132
	**R:** 5′-CGACCCGTCCTTTGAATT TC-3′	
*Bcl-2*	**F:** 5′ CAGATAGGCACCCAGGGTGAT 3′	114
	**R:** 5′ CATGTGTGTGGAGAGCGTCAAC 3′	
*Bax*	**F:** 5′-CTTTTGCTTCAG GGTTTCATCC-3′	165
	**R:** 5′-TTGAGACACT CGCTCAGCTTCT-3′	
*β-actin*	**F:** 5′-CCTGGCACCCA GCACAA-3′	198
	**R:** 5′-GCCGATCCAC ACGGAGTACT-3′	

### Statistical assay

In vitro data processing was achieved utilizing GraphPad Prism® version 5.00 (GraphPad Software, Inc., La Jolla, CA, USA) computer program and presented as means ± SD. One-way analysis of variance (ANOVA) followed by Tukey–Kramer multiple comparison test was employed to complete the stability data' statistical analysis. In vivo data were statistically analyzed with SPSS Statistics 22 (IBM Corporation, Armonk, NY, USA) based on ANOVA. Data are stated as the means ± SEM, and differences were measured significant when *P* < 0.05.

## Results

### Physicochemical Characterization of PRNL

**Figure 1:**
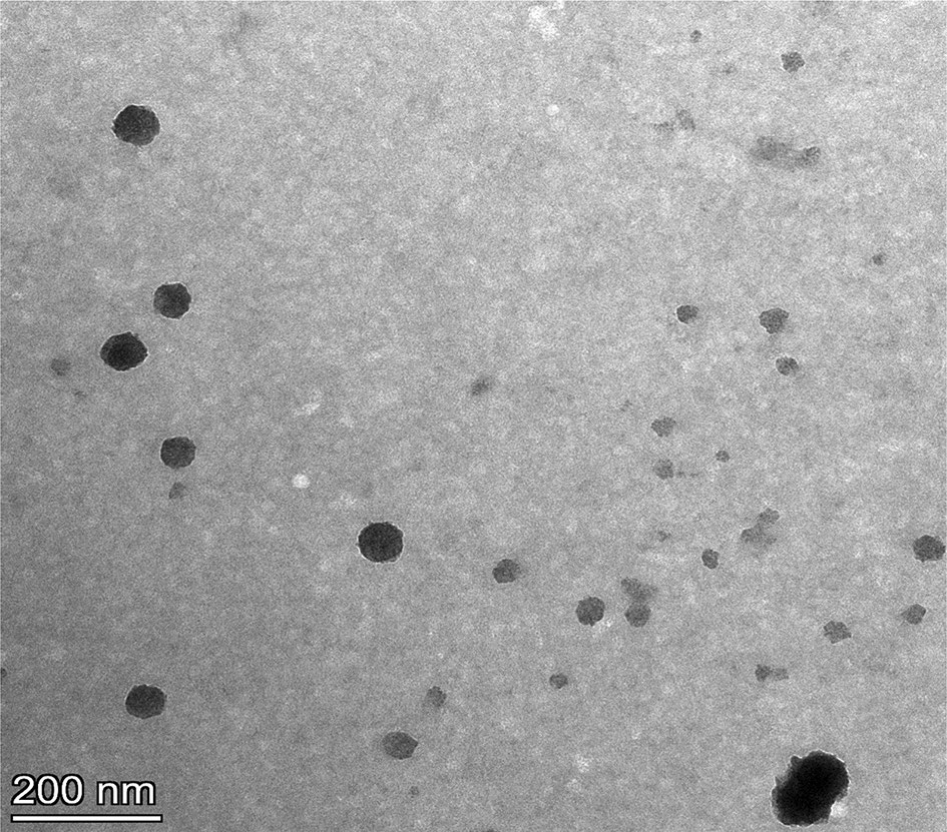
The characterization of TEM image showing a spherical morphology of PRNL with little or no aggregation observed.

The EE % was estimated to be 99.40% ± 0.036, stipulating that almost all PR was entrapped. The average ζP, D_h_, and PDI values of PRNL were found to be − 56.83 ± 1.27 mV, 113.13 ± 0.60 nm, and 0.380 ± 0.05, respectively. Imaging of the investigated PRNL dispersions revealed a spherical morphology with slight or no aggregation (Fig. 1).

**Figure 2:**
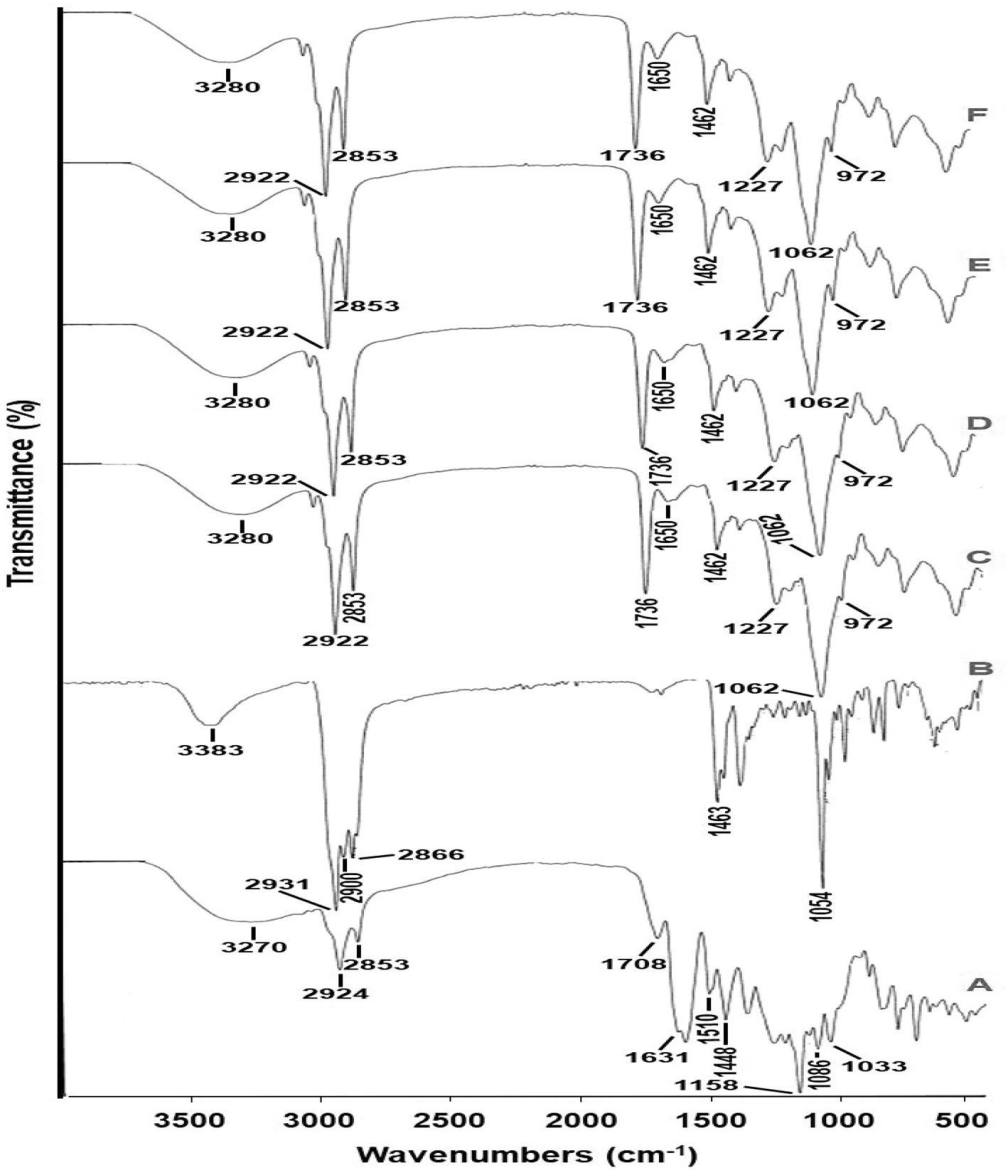
The FT-IR spectra of pure ethanol-extracted Chinese propolis (**A**), Cholesterol (**B**), Soybean lecithin (**C**), physical mixture of ethanol-extracted Chinese propolis, Cholesterol and Soybean lecithin (**D**), plain nanoliposomes (**E**) and ethanol-extracted Chinese propolis-loaded nanoliposomes (**F**).

As depicted in Fig. 2, the FT-IR spectrum of PR (A) depicts stretching of (O–H) group of phenolic molecules at 3270 cm^−1^. Besides, peaks at 2924 and 2853 cm^−1^ are distinguishing of C–H bending vibrations of (CH_2_, CH_3_) groups. The absorption bands at 1708, 1631, 1510 and 1448 cm^−1^ were accredited by the C=C of aromatic ring. The peaks at 1158, 1086 and 1033 cm^−1^ were ascribed by the extending vibration of the (C–O), (C–C) and (=C–O–C) bonds of flavonoids. In the Chol infrared spectrum (B), the stretching band of (O–H) group of phenolic compounds at 3383 cm^−1^ is depicted. The symmetric and asymmetric stretching vibrations of (–CH_2_, –CH_3_) groups are shown at 2931, 2900, 2866 and 1463 cm^−1^. The absorption band at 1054 cm^−1^ was due to aromatic ring disfigurement. In the IR spectrum of SL (C), the wide band at 3280 cm^−1^ could be ascribed to the existence of water. The symmetric and asymmetric extending vibrations of (–CH_2_) groups are depicted by the bands at 2922 and 2853 cm^−1^. Besides, the IR shoulders at 1736 and 1650 cm^−1^ represent ester carbonyl (C=O) group elongating vibrations, whereas that at 1462 cm^−1^ is related to scissoring vibrations of (–CH_2_) groups. Furthermore, the polar head groups vibrations are established by the existence of peaks at 1227 cm^−1^ typifying the phosphate (–PO_2_^−^) group asymmetric stretching, 1062 cm^−1^ typifying the (–PO_2_^−^) group symmetric stretching, as well as 972 cm^−1^ typifying the (–N^+^–CH_3_) antisymmetric extending vibrations. Physical mixture spectrum (D) displays the infrared bands of SL, while those of PR and Chol either appeared with diminished intensities, overlapped with those of SL or disappeared because of dilution factor. Similarly, NL_free_ (E) as well as PRNL (F) spectra were concurred with that of the physical mixture. In the contemporary study, neither sedimentation nor flocculation was observed, for the investigated PRNL preparation, during the appointed storage period at the various environments.

Table 2 outlines the detected numeric values of ζP, D_h_, PDI, and drug retention (%) for PRNL formulation, at the two various storage environments. For statistical comparison of the evaluated parameters during storage with those of the freshly prepared PRNL formulation, utilizing ANOVA, insignificant differences were detected upon storage at 4 ± 1 °C. Otherwise, only significant (*P* < 0.05) augment in D_h_ was clarified, while the other investigated variables exhibited insignificant inequality, upon storage at 25 ± 1 °C, 60 ± 5% RH.

**Table 2:** The values of ζP, PDI, D_h_ and drug retention (%) of PRNL aqueous dispersions stored at long-term storage conditions (4 ± 1 °C) as well as accelerated storage conditions (25 ± 1 °C, 60 ± 5% RH).

Evaluation parameters	Storage time^1^		
	Zero time	1 month	3 months
Long-term conditions (4 ± 1 °C)			
ζP (mV)	− 56.83 ± 1.27	− 56.13 ± 1.36	− 55.17 ± 2.12
PDI	0.380 ± 0.05	0.388 ± 0.02	0.391 ± 0.02
D_h_ (nm)	113.13 ± 0.60	112.17 ± 7.15	114.80 ± 1.31
Drug retention (%)	100.00 ± 0.00	99.63 ± 0.40	99.61 ± 0.57
Accelerated conditions (25 ± 1 °C, 60 ± 5% RH)			
ζP (mV)	− 56.83 ± 1.27	− 55.03 ± 1.36	− 53.93 ± 1.27
PDI	0.380 ± 0.05	0.400 ± 0.02	0.410 ± 0.02
D_h_ (nm)	113.13 ± 0.60	124.53 ± 2.75*	131.27 ± 1.40*^#^
Drug retention (%)	100.00 ± 0.00	99.67 ± 0.43	99.38 ± 0.16

^1^Data expressed as the mean ± SD (n = 3).

^#^Significant at *P* < 0.05 ambient conditions versus refrigeration after 3 months.

*Significant at *P* < 0.05 initial versus monthly.

### Effects of PRNL on sperm characteristics

The effects of various doses of PRNL supplemented with semen freezing extender on progressive motility, abnormality, membrane integrity, viability, and chromatin damage of buffalo bull spermatozoa after freeze-thawing are shown in Table 3. Supplementation with 2 or 4 µg/mL PRNL significantly (*P* < 0.05) increased sperm progressive motility, viability, and membrane integrity during semen cryopreservation As outlined in Table 3, adding high levels of PRNL (6 µg/mL) to the extender did not affect on most sperm attributes in compared with the control group. Interestingly, sperm abnormalities and the percentage of chromatin damages in spermatozoa were the lowest in the extender added 2 µg/mL PRNL (*P* < 0.05). The chromatin damage percentages did not differ significantly in post-thaw semen among treated groups; however, PRNL2 group produced a significant reduction when compared with the control (*P* < 0.05). Overall, both levels of PRNL (2 or 4 µg/mL) were the optimum levels for enhancing sperm functionality during semen cryopreservation. Additionally, higher levels of PRNL (6 µg/mL) did not provide further full patronage to sperm form cryodamage.

**Table 3:** Effect of propolis-loaded nanoliposomes (PRNL) on progressive motility, viability, membrane integrity, abnormality and chromatin damage of buffalo bull spermatozoa during cryopreservation (Means ± SE).

Semen attributes (%)	Treatments^1^			
	PRNL0	PRNL2	PRNL4	PRNL6
Progressive motility	38.0 ± 1.22^b^	56.0 ± 1.87^a^	54.0 ± 1.00^a^	41.0 ± 1.87^b^
Viability	42.0 ± 1.48^b^	57.4 ± 1.63^a^	55.2 ± 0.86^a^	45.0 ± 1.64^b^
Membrane integrity	45.4 ± 1.86^b^	59.4 ± 1.66^a^	56.4 ± 1.63^a^	45.6 ± 1.86^b^
Abnormality	16.6 ± 1.57^ab^	12.8 ± 1.02^b^	17.8 ± 1.28^ab^	18.4 ± 1.33^a^
Chromatin damage	7.8 ± 0.49^a^	5.6 ± 0.51^b^	7.4 ± 0.60^ab^	7.4 ± 0.40^ab^

^1^Freezing extender fortified with various levels of propolis-loaded nanoliposomes (PRNL) containing 0 (PRNL0), 2 (PRNL2), 4 (PRNL4) and 6 (PRNL6) µg/mL, respectively.

^a, b^means represented within the same row with various superscripts are significantly different at *P* < 0.05.

### Effects of PRNL on antioxidant and oxidative stress

As depicted in Table 4, the addition of PRNL in the buffalo sperm extender media exhibited significant (*P* < 0.05) impact on antioxidant indices (TAC, GPX, CAT and SOD activities), as well as oxidative variables (ROS, and MDA concentrations). The PRNL2 group exhibited the best results for all anti-oxidative activities with significantly higher levels than the other groups (*P* < 0.05). The TAC values in both groups PRNL2 (10.6 ± 1.37) and PRNL4 (3.3 ± 0.06) were significantly higher (*P* < 0.05) than the PRNL6 (1.3 ± 0.04) and control (0.9 ± 0.07) groups. The TAC activity in the PRNL2 group was greater than that in the other experimental groups (*P* < 0.05), but no statistical differences were observed among the PRNL4, PRNL6 and the control group (*P* > 0.05). For the GP_X_, CAT and SOD values, the best results were achieved with PRNL2 group, followed by PRNL4 group then PRNL6 (Table 4). The PRNL2 exhibited the lowest values of ROS and MDA in comparison with other treated groups. There was no significant change in ROS levels between both PRNL4 and PRNL6 treated groups (*P* > 0.05). In synopsis, compared with the control group, the addition of 2 µg/mL PRNL to the freezing extender of buffalo sperm significantly augmented the TAC, GP_X_, CAT, and SOD values and significantly decreased the concentrations of MDA and ROS.

**Table 4:** Effect of propolis-loaded nanoliposomes (PRNL) on antioxidant and oxidative markers in extender of post-thawed buffalo bull semen (means ± SE).

Measurements^1^	Treatments^2^			
	PRNL0	PRNL02	PRNL4	PRNL6
Antioxidative markers				
TAC (ng/mL)	0.9 ± 0.07^b^	10.6 ± 1.37^a^	3.3 ± 0.06^b^	1.3 ± 0.04^b^
GPx (U/mL)	41.8 ± 3.32^d^	194.7 ± 3.51^a^	118.3 ± 3.40^b^	78.1 ± 1.83^c^
CAT (ng/mL)	1.1 ± 0.06^d^	12.2 ± 0.55^a^	5.0 ± 0.20^b^	2.5 ± 0.19^c^
SOD (U/mL)	18.3 ± 0.42^d^	245.5 ± 5.67^a^	107.3 ± 3.45^b^	63.8 ± 2.52^c^
Oxidative markers				
ROS (U/mL)	745.0 ± 27.87^a^	226.7 ± 7.13^c^	368.3 ± 15.45^b^	440.3 ± 17.07^b^
MDA (nmol/mL)	11.8 ± 0.33^a^	1.4 ± 0.25^d^	4.7 ± 0.36^c^	7.8 ± 0.29^b^

^1^MDA, Malondialdehyde; GPx, Glutathione Peroxidase; TAC, Total Antioxidant Capacity; SOD, Super Oxide Dismutase; CAT, Catalase; and ROS, Reactive Oxygen Species.

^2^Freezing extenders fortified with various levels of propolis-loaded nanoliposomes (PRNL) containing 0 (PRNL0), 2 (PRNL2), 4 (PRNL4) and 6 (PRNL6) µg/mL, respectively.

^a–d^Means represented within the same row with various superscripts are significantly different at *P* < 0.05.

### Sperm apoptosis indicator

**Figure 3:**
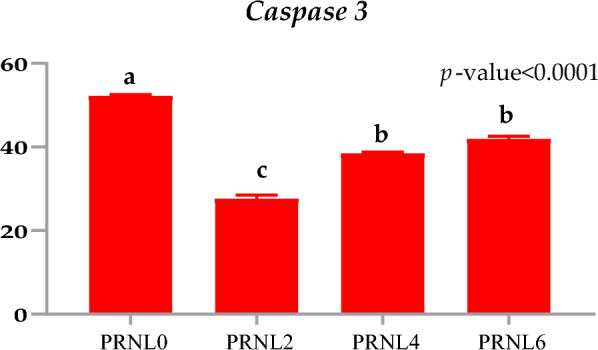
The values of Caspase 3 in spermatozoa of Egyptian buffalo bull treated with different levels of propolis-loaded nanoliposomes (PRNL) containing 0 (PRNL0), 2 (PRNL2), 4 (PRNL4) and 6 (PRNL6) µg/mL, respectively. ^a-c^Means represented within the same column with various superscripts are significantly different at *P* < 0.01.

To explore the beneficial effects of PRLN on sperm apoptosis, caspase content in the sperm were assessed (Fig. 3). As shown in Fig. 3, the addition of PRLN effectively reduced caspase 3 (as an apoptosis indicator) in the buffalo spermatozoa at cryopreservation, particularly when the extender supplemented with 2 µg/mL of PRNL (*P* < 0.001). The addition of 4 or 6 µg/mL PRNL exhibited significantly lower *Caspase 3* and there was no statistical alteration in Caspase 3 between both PRLN4 and PRLN6 groups (*P* > 0.05), when compared with the PRNL0 group.

### Effects on apoptotic genes in spermatozoa

**Figure 4:**
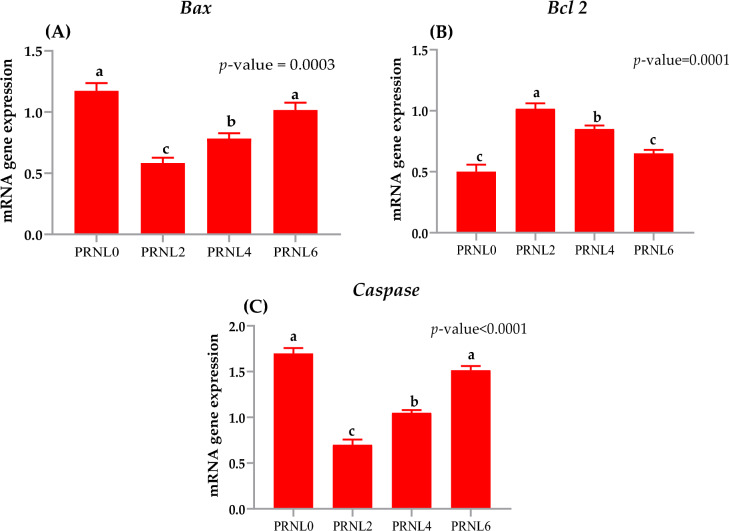
Effects propolis-loaded nanoliposomes (PRNL) on mRNA expression of apoptotic related genes in spermatozoa including (**A**) pro-apoptotic *BAX* (BCL2-associated X), (**B**) anti-apoptotic *BCL-2* (B-cell lymphoma), and (**C**) Caspase 3 using RT-qPCR. Tris extender fortified with different levels of propolis-loaded nanoliposomes (PRNL) containing 0 (PRNL0), 2 (PRNL2), 4 (PRNL4) and 6 (PRNL6) µg/mL, respectively the values have different letters (a, b, and c) are significantly different at *P* < 0.05.

Figure 4A–C display the transcript levels of apoptosis-related proteins as confirmed via RT-qPCR. Supplementing with 2 µg/mL of PRNL produced a considerable decline in the pro-apoptotic mRNA expression of *Bax* (Fig. 4A) and caspase 3 (Fig. 4C) genes in the sperm (*P* < 0.003), followed by PRN4 group, while there was no significant change between the control and PRNL6 groups (*P* > 0.05) for *Bax* and caspase 3 genes. In contrast, the adding of 2 or 4 µg/mL PRNL resulted in a significant upregulated in the transcript of the anti-apoptotic gene Bcl-2 (*P* < 0.001). The PRNL4 exhibited intermediate values for Bcl-2 with significant difference. No significant alterations were noticed among the PRNL0 and PRNL6 groups for Bcl-2 gene in spermatozoa (*P* > 0.05). Overall, PRNL supplementation (especially, 2 or 4 µg/mL) to the freezing media of buffalo spermatozoa significantly reduced the apoptosis-related genes (*Bax* and caspase 3), while significantly improved the anti-apoptosis-related gene *Bcl-2* in the buffalo sperm (Fig. 4B).

### Sperm ultrastructure

**Figure 5:**
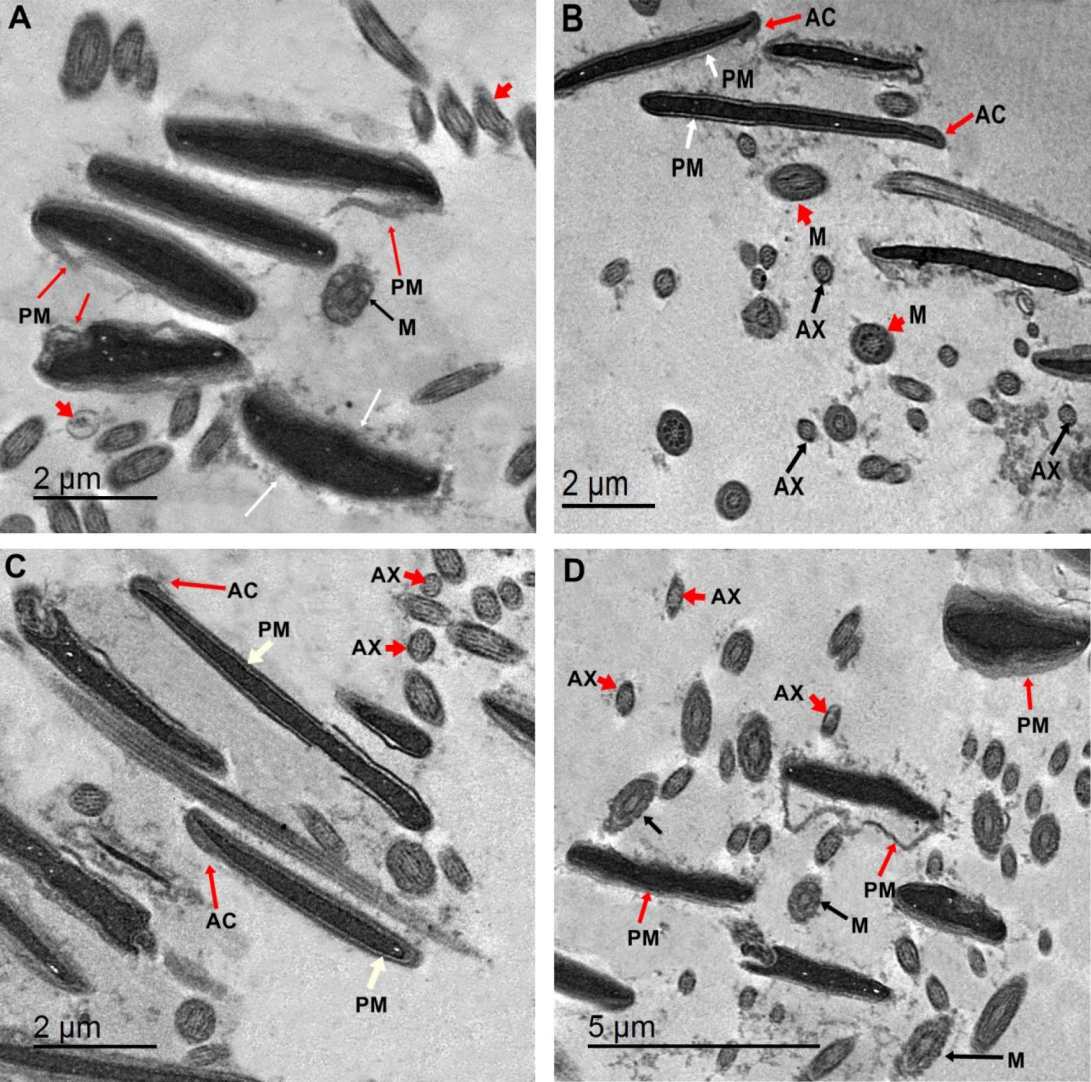
Effects propolis-loaded nanoliposomes (PRNL) on post-thawed sperm ultrastructure. Extender supplemented with different levels of PRNL containing 0 (**A**), 2 (**B**), 4 (**C**) and 6 (**D**) µg/mL, respectively. Plasma membrane in the sperm heads of PRNL0 group were showing loosening, swelling and breaking. Moreover, vesicular-swollen mitochondria, tail coiling, and marginated disrupted chromatin, irregular and heterogeneous shape were observed in the PRNL0 group (**A**). The addition of 2 (PRNL4) or 4 (PRNL6) presented less damages in the nucleus, plasma membrane and sperm neck or tail (**B**,**C**). The addition 2 μg/mL of PRNL (PRNL2 group) was more effective in diminishing the rupture rates of acrosomal and plasma membranes, and sperm tail after freeze-thawing in related to the other groups. The buffalo semen treated with 6 μg/mL PRNL (D) revealed high rate of injured/absent acrosome and plasma membranes, demonstrating that the high doses of PRNL botched to sustain the fine ultrastructure of sperm. PM (Plasma membrane), AC (Acrosome), Mitochondria (M) and Axon of tail (AX).

For more insight and better comprehend on the subcellular structure changes of spermatozoa after freezing and thawing, we accomplished TEM screening of the structural impairment in buffalo spermatozoa (Fig. 5A–D). As shown in Fig. 5A, the buffalo sperm showed absence of plasma membrane, and damage tail and neck, acrosome disruption, and altered acrosome membranes. The addition of 2 (Fig. 5B) or 4 μg/mL PRNL (Fig. 5C) showed less damages in the nucleus, acceptable integrity of acrosome and plasma membranes. The addition 2 μg/mL PRNL (PRNL2 group) was more effective in diminishing the rupture rates of acrosomal integrity, plasma membrane, and sperm tail after post-thawing in relative to the other groups. The buffalo semen treated with 6 μg/mL PRNL (Fig. 5D) discovered superior degree of injured/ indistinct acrosome and plasma membranes, demonstrating that the greater levels of PRNL futile to sustain the fine ultrastructure of spermatozoa.

## Discussion

Sperm cryopreservation is a robust way for protective male genetic resources in liquid nitrogen for many years until uses. The merging system of sperm cryopreservation and artificial insemination technique can accomplish hybrid enhancement, thus escalating the number of admirable diversities. However, the accretion of unwarranted ROS during cryopreservation causes sperm cryodamage’s in buffalo. For this reason, freezing media fortification or manipulation with powerful antioxidants could reduce the determinately effects of cryodamage’s of sperm^2,9^. In this research, we explored the protective ability of propolis-loaded nanoliposomes (PRNL), for the first time, as a novel antioxidant and anti-apoptotic mediator during the sperm cryopreservation of buffalo. The results exhibited that PRNL significantly enhanced the sperm progressive motility, viability, plasma membrane integrity, and the activities of TAC, CAT, SOD, and GPx of freeze-thawed buffalo sperm while meaningfully declining the chromatin damages, abnormalities percentages as well as ROS and MDA amounts. The best level of PRNL as determined in this study was arranged between 2 and 4 µg/mL. Furthermore, at apoptosis transcriptomic level, PRNL protected buffalo sperm during cryopreservation by improving the expression of anti-apoptotic gene *Bcl2*, impeding the expression of apoptotic genes *Caspase 3* and *Bax*, and decreasing the *Caspase 3* activity in spermatozoa.

Efficiency in entrapment is an last matter in case of implementing NLPs as a nanocarrier^30^. To discriminate between misguided PR privileged NLPs and the unentrapped one that was outside, ultrafiltration of the subsequent yellow translucent colloidal dispersion was carried out as reported recently^31^. Thereafter, unentrapped PR could be detected spectrophotometrically at 296 nm, in the ultrafiltrate, without more extension. The EE % was found to be 99.40% ± 0.036 stipulating that almost all PR was entrapped. The achieved high EE% of PR was even higher than that obtained when^30^, encapsulated PR in liposomes, that consequently ensure effective and convenient delivery of such biomolecule in the recommended levels. Such obtained results insinuated that NLPs are appropriate for PR encapsulation with superior EE % which is an iterative demand to decline both the manufacturing cost and the consumed carrier materials, while little nano-formulation of remedy will be applied to supplement the essential therapeutic dosage^32^.

Regarding nanoparticulate drug delivery systems, not only their D_h_ values but also PDI ones, known to express the average uniformity of a particle solution, are parameters which have an important impact on bioavailability, biodistribution as well as colloidal dosage form stability upon storing. It can be inferred that the small D_h_ of PRNL might concentrate them capable to penetrate sites such as such as the plasmatic membrane of spermatozoa and could protect this component form damages induced by cryopreservation process. Likewise, small PDI values indicate narrow particle size distribution, imply homogeneously arranged figurations, and ensure enhanced colloidal stability without creation of ingathering. The attainable spherical morphology of PRNL (Fig. 1), with slight or absence aggregation might be ascribed to the superior negative ζP value. Furthermore, boosted colloidal stability of the formulated NLPs under physiological situations might be surmised^33^.

Concerning the FT-IR spectra (Fig. 2), the previously noticed peaks obviously characterize the chemical nature of pure PR, however a minor modification of its specific peaks, when compared to those described in the literatures, could be attributed to the various source of propolis^34,35^. Besides, matched peaks were documented previously for Chol^36^ and SL^37,38^. PRNL spectrum illustrated PR entrapment in the synthesis NLPs. Generally, NLPs’ shelf-life stability is determined via evaluating the pivotal variables primarily; physical attendance, ζP, D_h_, PDI as well average drug retention (%), which evaluate their chemical and physical stabilities^19^.

In the current survey, PRNL formulations exhibited neither flocculation nor sedimentation upon storage for a period of three months at the two various studied situations, stipulating high physical stability of the prepared NLPs system. Sperm motility is a good indicator for sperm functionality toward fertility. As well known, sperm exposed to cold shock or cryopreservation showed significant reduction in sperm quality and functionality^2^. Moreover, the cryopreservation process is found to have unfavorable impacts on the sperm function and structure. In this sense, the addition of an effective antioxidant could protect this influence and enhance the sperm motility^4,6^. Studies have found that the freezing media fortified with nanoparticles enhanced significantly motility of post-thawed sperm of rabbit^5^, bull^21^, rooster^9^ and ram^25^. While results about using the PRNL in freezing media are lacking. In the other line, many previous works^10,11,13–17^ clarified the protective role of propolis in enhancing the sperm motility, viability, and membrane integrity after cryopreservation in different animal species and fish. Moreover, a significant reduction in the percentages of abnormalities and chromatin damages were observed in our study as similar to previous works^11,13,16^. This improvement in semen quality might ascribe to the potential antioxidant efficiency of new form of PRNL added to semen extender. As we explored previously, the nanoliposome is more effective method for targeting and could enhance the stability, solubility and availability, then it could make a film around the sperm and protect them from cryoinjury^39,40^. By fortification the antioxidant PRNL in the current investigations, the beneficial effects of PRNL augmented the superiority of sperm function and boosting the sperm protection which can be used in buffalo cryopreserved semen. It’s of interesting to develop a new freezing media for promoting the artificial insemination of buffalo as well improving the reproductive efficiency of this species. The existing verdicts discovered that PRNL had some promising effects on sperm motility and plasma membrane and acrosomal integrities. These valuable characteristics can be accredited by the critical function that PRNL in boosting antioxidant capability, which is vital mediator in conserving the plasma membrane of sperm against oxidative stress. This feature was confirmed by significantly higher levels of acrosome integrity and viability in sperm with the supplementing of PR to the freezing extender, which in turn produced in enhancements in post-thaw sperm function^11,13,16,17^. So, it seems that some constructive impacts of PRNL on sperm motility may be owing to sustaining the plasma membrane integrity. Supplementing 2 or 4 μg/mL of PRNL into freezing media of buffalo sperm showed best results for sperm motility, viability, acrosome and plasma membrane integrities as well as declining the abnormal sperm.

Accessible evidence exhibits that the cryopreservation process induced a substantial diminution in antioxidant system, which make the sperm more vulnerable to lipid peroxidation and thus high amounts of ROS are generated. Despite the physiological levels of ROS are biologically perform critical roles in mostly of cellular pathways, the high amount causes imbalance the defensive system, DNA, protein and lipid damages in sperm^4^. Countersting the high generated ROS form freezing media can a robust strategy for enhancing the sperm functionality and protect the sperm from cryoinjury^1^. Our data found that the addition of freezing media with PRNL (2 or 4 μg/mL) reduced significantly the ROS, and MDA as well as significantly improved the antioxidant enzymes such TAC, GPx, CAT and SOD levels. In other words, the freezing media enriched with propolis produced a considerable effect in lipid peroxidation inhibition, and oxidative stress scavenging ability, which has been previously reported by several authors^10,11,13,15^. However, we did not find any previous works in related to addition of PRNL in the freezing extender, whereas the majority of previous studies were conducted on propolis. To our knowledge this is the first exploration on the using of PRNL fortified in freezing extender of buffalo semen. This feature could be due to its highly contents of a large number of antioxidative molecules such as ellagic, caffeic, ferulic syringic acids, and quercetin as previously scrutinized by HPLC and LC–MS/MS^41^. According to^11^, the addition of propolis (25–50 μM) in freezing extender of bull semen produced significantly superior in TAC amounts. Moreover, Soleimanzadeh et al.^42^ indicated that the caffeic acid (50–100 μM) decreased MDA levels and significantly increased TAC and GSH activities after freezing–thawing in bull semen, which are similar with our data presented in this research. Caffeic acid is one of the chief ingredients of propolis with robust antioxidant capacity.

Our verdicts confirmed that fortification with PRNL supported ROS scavenging action while simultaneously decreasing the cell death or apoptosis. Moreover, the PRNL administration inhibited cell death via decline ROS creation, consequently regulatory the opening of mitochondrial permeability transition pores and sustaining regular cellular events. In this study, we lack the studying of PRNL on the mitochondria activity of post-thawed buffalo. The obtained findings demonstrated the fortifications of PRNL (as anti-apoptotic mediator) with freezing media decreased the number of apoptotic sperm formed after thawing. Propolis loaded nanoliposomes (RPNL at 2 μg/mL) exhibited significantly upregulation of *Bcl2* gene and downregulation of *capase3* and *Bax* genes. In contrast with our results, Azarshinfam et al.^43^ found that the propolis nanoparticles induced significant an upregulation in *Bax* gene, and downregulation in *Bcl-2*, thus inducing apoptosis, which supporting its efficacy in treating colorectal cancer. According to reports from^11,15,16^, propolis exerts its cryoprotective benefits by maintaining redox homeostasis and decreasing apoptosis in semen cryopreserved. Najafi et al.^39^ described that the addition of lycopene-loaded nanoliposomes (0.2 mM) showed significantly reduction in the percentage of apoptotic sperm in rooster post-thawed semen. Moreover, based on the previous work of used ellagic acid-loaded liposomes (1 mM)^44^ and quercetin loaded liposomes (15 μM)^40^ significantly improved the antioxidant indices and reduced the apoptotic sperms in post-thawed rooster sperm. The consequences of the existing research clarified that the fortifications of PRNL to the semen extender significantly diminished the capase3 enzyme in sperm after thawing. Moreover, detecting the apoptosis marker is valuable verdicts for assessing the sperm health and functionality after cryopreservation. In this study, we have developed a new form of liposomes which have been implied to enhance the competence of some hydrophilic and lipophilic antioxidants. As mentioned before, liposomes may augment thermal, water solubility, and pH stability of bioactive molecules, because the encapsulation offers a physical barrier toward sperm against cryodamage^1^. In some studies, it was found that the propolis regulated the transcripts of *Bax* and *Bcl-2* avoiding activation of the mitochondrial apoptotic paths in sperm^10,13^, therefore declining apoptotic sperm which evidenced in the current observation. The valuation of post-thaw viability and apoptosis status shown that the fortification of PRNL to the freezing media improved the number of sperm viability, whereas its simultaneously diminishing the apoptotic, abnormalities and dead sperm.

For more insight on the cryoprotective defense of PRNL in buffalo post-thawed semen, we investigated the influences of PRNL on the ultrastructure changes of sperm which is performed by TEM. Based on the data in this research, addition of PRNL to the freezing media of buffalo semen could maintain the sperm functionality and health through diminishing the breakage rates of acrosomal nuclear membrane, acrosomal damages, plasma membrane, vesicular-swollen mitochondria, sperm tail coiling and marginated disrupted chromatin as detected in all treated groups. Moreover, the plasma membrane of sperm faithfully bounded the nucleus, middle piece, and flagellums were demonstrated in the PRNL6 after thawing. Our data in accordance with aforementioned experiments^5,21^, who found that the supplementation of nanoparticles to the freezing extender sustain the sperm ultrastructure and improved sperm functionality in bull and rabbit, respectively.

Conferring to the current data, the augmented viability was utmost probable the result of antiapoptotic action of PRNL, which in turn resulted in augmented velocity and motility of the sperm. Many reports have been confirmed that the excess amounts of OS triggered to damage plasma membrane during the freeze–thaw process which is harmfully related to post-thaw motility, viability, and ultimately fertilization competence^4,21^. Freezing extender fortified with PRNL strengthens sperm motility, viability, and sustained plasma membrane functionality which subsequently boosted the antioxidative defense, reduced the apoptotic genes and sustain the sperm ultrastructure. This a new type of phytochemical loaded nanoliposome may open a new window for enhancing the freezing protocol in several spices, which in turn improving the reproductive efficacy and fertility in farm animals.

## Conclusion

Propolis loaded nanoliposomes at 2 or 4 µg/mL could be an appropriate supplement mediators for cryopreservation of buffalo sperm, due to its ability to improve post-thawed buffalo sperm quality comprising motility parameters, antioxidant status, and reducing the oxidative stress, and apoptotic status, totally result in higher freeze ability of buffalo sperm. More studies should be investigated to explore these cryoprotective effects of PRNL in buffalo cryopreserved semen especially at the transcriptomics, proteomics and lipidomic levels.

## Data Availability

All authors declare that the data supporting the findings of this study are available within the paper.
